# Circulating microRNAs as Potential Biomarkers of Infectious Disease

**DOI:** 10.3389/fimmu.2017.00118

**Published:** 2017-02-16

**Authors:** Carolina N. Correia, Nicolas C. Nalpas, Kirsten E. McLoughlin, John A. Browne, Stephen V. Gordon, David E. MacHugh, Ronan G. Shaughnessy

**Affiliations:** ^1^Animal Genomics Laboratory, UCD School of Agriculture and Food Science, University College Dublin, Dublin, Ireland; ^2^UCD School of Veterinary Medicine, University College Dublin, Dublin, Ireland; ^3^University College Dublin, UCD Conway Institute of Biomolecular and Biomedical Research, Dublin, Ireland

**Keywords:** biomarker, diagnostic, infection, transcriptomics, microRNA, serum, plasma

## Abstract

microRNAs (miRNAs) are a class of small non-coding endogenous RNA molecules that regulate a wide range of biological processes by post-transcriptionally regulating gene expression. Thousands of these molecules have been discovered to date, and multiple miRNAs have been shown to coordinately fine-tune cellular processes key to organismal development, homeostasis, neurobiology, immunobiology, and control of infection. The fundamental regulatory role of miRNAs in a variety of biological processes suggests that differential expression of these transcripts may be exploited as a novel source of molecular biomarkers for many different disease pathologies or abnormalities. This has been emphasized by the recent discovery of remarkably stable miRNAs in mammalian biofluids, which may originate from intracellular processes elsewhere in the body. The potential of circulating miRNAs as biomarkers of disease has mainly been demonstrated for various types of cancer. More recently, however, attention has focused on the use of circulating miRNAs as diagnostic/prognostic biomarkers of infectious disease; for example, human tuberculosis caused by infection with *Mycobacterium tuberculosis*, sepsis caused by multiple infectious agents, and viral hepatitis. Here, we review these developments and discuss prospects and challenges for translating circulating miRNA into novel diagnostics for infectious disease.

## What Makes a Good Biomarker?

According to the working group of the National Institutes of Health Director’s Initiative on Biomarkers and Surrogate Endpoints, a biomarker is “a characteristic that is objectively measured and evaluated as an indicator of normal biological processes, pathogenic processes, or pharmacologic responses to a therapeutic intervention” (1). A simpler but broader definition of biomarkers as objective, quantifiable characteristics of biological processes has also been emphasized by Strimbu and Tavel (2). The ideal biomarker has high specificity and sensitivity, is detectable by minimally invasive sampling procedures, and its concentration should be indicative of a disease state (1–5). Diagnostic biomarkers can be used to evaluate disease status, prognostic biomarkers are informative of disease outcome, and predictive biomarkers help determine treatment efficacy when experimental groups are compared to controls (4, 6).

In recent years, high-throughput sequencing (HTS) technologies have enabled simultaneous screening of thousands of potential transcriptional biomarkers, which facilitates both discovery of specific host disease expression biosignatures (7–9) and new insight on host–pathogen interaction and immunobiology (10–12). Host biomarkers may also help evaluate vaccine efficacy in both humans and domestic animals (13, 14), as well as provide information on the molecular mechanisms underlying latent infections (15) and drug resistance in pathogens (16).

## Canonical Biogenesis and Immunological Functions of microRNAs (miRNAs)

The role of miRNAs in post-transcriptional regulation of gene expression was discovered in 1993 through analyses of the *lin-4* locus in the roundworm *Caenorhabditis elegans*. Two contemporaneous studies showed that an RNA transcript from *lin-4* repressed translation of the lin-14 messenger RNA (mRNA), thereby exerting temporal developmental control on a diverse range of cell lineages (17, 18). Since then, it has been demonstrated that eukaryotic organisms contain hundreds to thousands of these small non-coding regulatory RNA molecules (19). Many miRNAs are evolutionarily conserved across divergent metazoan taxa (20, 21), highlighting the extensive roles that these small RNAs play in the regulatory networks and pathways governing complex biological processes such as cell fate specification, and innate and adaptive immunity (22–24).

Canonical biogenesis of miRNA in mammalian cells starts with transcription of a long RNA molecule called the primary-miRNA (pri-miRNA) by RNA polymerase II (25). Within the nucleus, pri-miRNA undergoes cleavage by the microprocessor complex, which consists of a Drosha ribonuclease III and the RNA-binding DGCR8 microprocessor complex subunit protein (26, 27). The intermediate product is a precursor-miRNA (pre-miRNA) hairpin of ~70 nucleotides in length that is transported to the cytoplasm by the exportin-5 protein (28). An additional cleavage occurs near the pre-miRNA terminal loop through the action of endoribonuclease Dicer (29). The final product is an 18–25 nucleotide double-stranded RNA with short 3′ overhangs that binds to argonaute (AGO) proteins and is loaded into the RNA-induced silencing complex (RISC) by the RISC-loading complex (RLC), which is formed by endoribonuclease Dicer, RLC subunit TARBP2, and AGO1–4 proteins (30). One strand of the RNA duplex, the mature miRNA, remains within the RLC and is used as a guide by the RISC for complementary nucleotide base pairing with a target mRNA (31). The second strand is known as miRNA* (or passenger strand) and is normally degraded after its release from the RLC. Further details on canonical biogenesis (32) and the processes driving mature miRNA strand selection (33, 34) have been extensively reviewed elsewhere. The development of HTS technologies has facilitated high-resolution miRNA-sequencing (miRNA-seq), revealing the existence of multiple functional mature variants that are termed isomiRs (35–37). In addition, non-canonical pathways have been identified as alternative mechanisms of miRNA biogenesis (38, 39).

Dysregulation of intracellular miRNAs during disease was first reported in 2002, with evidence that miR-15 and miR-16 were tumor suppressors for chronic lymphocyte leukemia (40). Shortly afterward, it was shown that higher let-7 expression levels were associated with a better prognosis for lung cancer survival (41). Notably, the cancer research literature has highlighted miRNAs as powerful classifiers for disease onset and patient survival (42–45), as well as tumor driver mutations (46–48). These, and several other studies that followed, laid the groundwork for research that focuses on exploring the potential of miRNAs as biomarkers and therapeutic gene targets.

*In silico* analyses suggest that at least two-thirds of mammalian mRNAs are regulated by miRNAs (22, 23, 49); therefore, it is perhaps unsurprising that these non-coding transcripts have emerged as important molecular fine-tuners of the host immune response during infection (50–54). For example, multiple miRNAs are known to regulate the toll-like receptor 4 (TLR-4) pathway in the host innate immune response (23, 55, 56) and are also essential for optimal T cell activation and differentiation (57–62). More specifically, mice lacking miR-155 show diminished immune responses against infections with *Citrobacter rodentium* (63), *Salmonella typhimurium* (64), and *Listeria monocytogenes* (65). miR-155 has also been found to be increased in peripheral monocytes of chronic hepatitis C (CHC)-infected patients following *in vitro* stimulation with lipopolysaccharide (LPS) (66), and in murine bone marrow-derived macrophages stimulated with LPS plus interferon-γ (IFN-γ) (67). miR-146 is another important miRNA that exhibits increased expression in immune cells following TLR activation by bacterial pathogens (68). Moreover, members of the miR-146 family were found to form distinct expression profiles in human monocyte-derived macrophage cells infected with *Mycobacterium tuberculosis* (69) and *M. bovis*-infected bovine alveolar macrophages (70). The immunoregulatory roles of miRNAs in different cells involved with the host response to bacterial infections has been comprehensively reviewed (71, 72).

Collectively, these studies highlight the importance of post-transcriptional regulation of gene expression mediated by intracellular miRNAs in mammalian infection and immunity processes. A growing number of public databases provide information on miRNA–disease relationships (73), and informative reviews on this topic have been published (22, 23, 49, 52, 74).

### Circulating miRNAs

**Figure 1:**
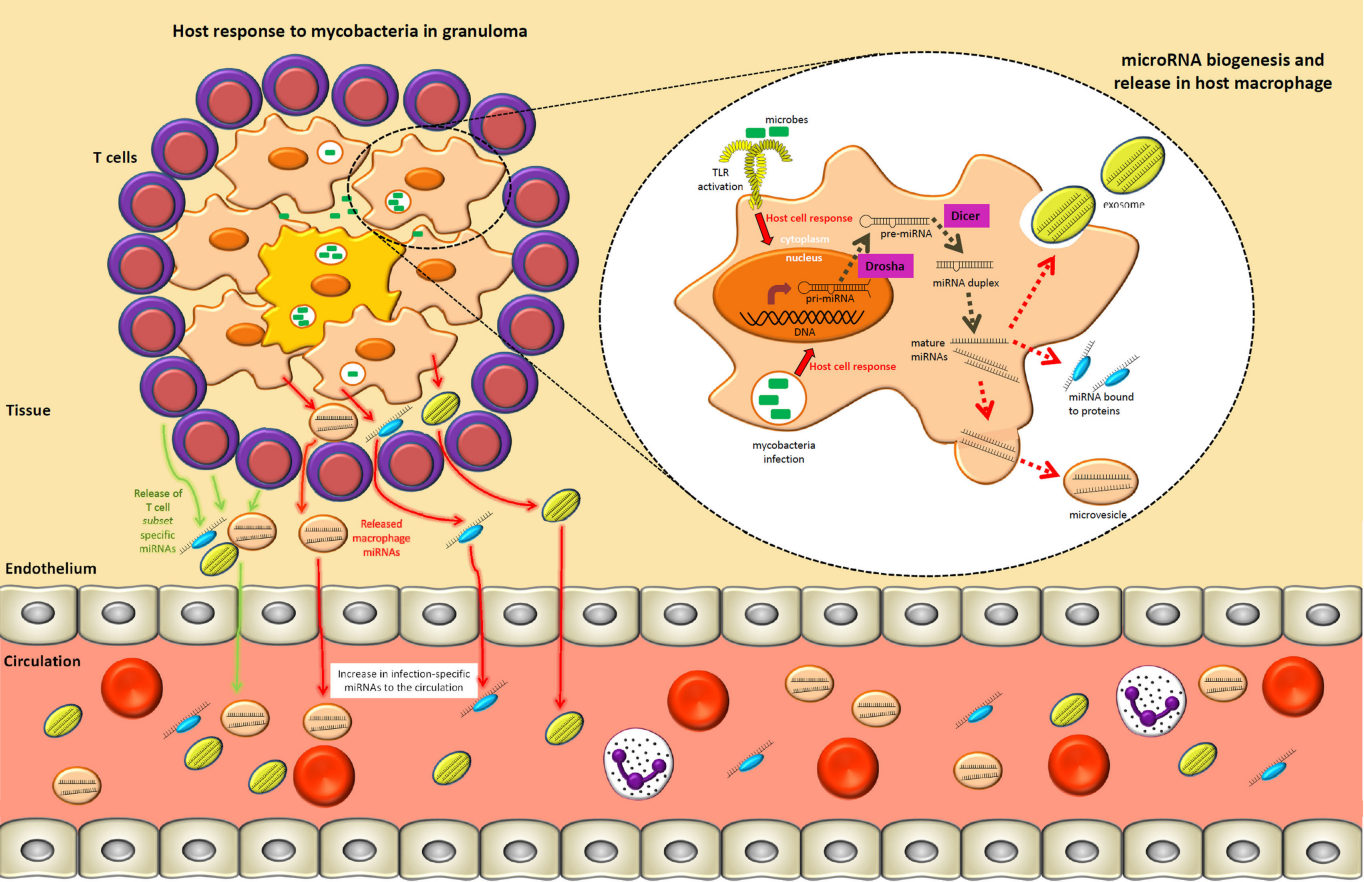
**A tuberculosis lung granuloma demonstrates how specific circulating microRNAs (miRNAs) may arise during an infection process**. Mycobacterial pathogen-associated molecular patterns are recognized by toll-like receptors (TLRs) and other pattern recognition receptors, which result in the upregulation of primary-miRNAs in macrophages. These transcripts are subsequently cleaved in the nucleus and cytoplasm by Drosha and Dicer, respectively, resulting in 21–25 nucleotide mature miRNAs that act to fine-tune intracellular immune processes. Specific pathways and components of the immune response may be regulated by different miRNA subsets. Concurrently, the surrounding T lymphocytes involved in granuloma formation/maintenance upregulate T cell subset-specific miRNAs as a means of modulating the type of adaptive immune response. Mature miRNAs generated in macrophages and T cells may also be released into the extracellular environment within exosomes, heterogeneous microvesicles, or in association with high-density lipoprotein, LDL, or other protein complexes. Subsequently, by means not yet fully understood, these extracellular miRNAs move from local sites of infection to the circulatory system. This process can therefore give rise to infection-specific circulating miRNA expression signatures that can readily be accessed from multiple biological fluids (e.g., serum, plasma, or sputum).

So far, we have shown examples of intracellular miRNAs with immunological roles; however, there is a growing consensus that immune and non-immune cells routinely and actively release miRNAs into extracellular environments (75–77). Commonly associated with RNA-binding proteins, high-density lipoprotein particles or enclosed within lipid vesicles (Figure 1), miRNAs have been found to be extremely stable in extracellular fluids of mammals, such as blood plasma, serum, urine, saliva, and semen (78–80). miRNAs released by a human THP-1 monocyte cell line may be taken up by recipient cells in an alternative means of cell-to-cell communication (81). Wang and colleagues have shown that nucleophosmin, an RNA-binding protein involved with nuclear export of ribosomes, mediates export and protection of circulating miRNAs against degradation in several human cell lines (HepG2, A549, T98, and BSEA2B) immediately after serum deprivation, which is suggestive of an active response to stress (82). Active release of extracellular circulating miRNAs supports the hypothesis that they may act as “hormones” in cell-to-cell communication (82, 83).

Further work is required to fully understand how the release of extracellular miRNAs and uptake by target cell populations influences biomolecular signaling networks. Regardless of their precise functions, the main utility of miRNAs in the field of diagnostics and prognostics is based on the premise that different miRNA expression signatures are linked to different pathological states (Figure 1). With this in mind, it is noteworthy that a number of infectious diseases have been the focus of recent studies to assess circulating miRNAs as biomarkers.

## Challenges for Accurate Detection of Circulating miRNAs in Biofluids

The observation that extracellular nucleic acids (both DNA and RNA) are present in vertebrate bodily fluids was first recorded almost 70 years ago (84), but their potential as biomarkers for disease states was not fully realized until the 1990s (85). In turn, detection of extracellular miRNAs was first reported in 2008 when placental miRNAs were observed in maternal plasma (86). In the same year, circulating miRNAs were also described in blood serum (87, 88) and plasma samples collected from cancer patients (88). In this regard, the potential of circulating miRNAs as non-invasive diagnostic and prognostic biomarkers of disease status in biological fluids was first realized in the field of cancer biology, particularly because techniques for cancer diagnosis and prognosis still primarily rely on invasive tissue biopsies (89–91), and the establishment of new circulating protein biomarkers has not been able to meet the demand (92). The marked stability of circulating miRNAs in body fluids, which are still viable after repeated cycles of freeze–thawing and long-term storage of frozen samples (88, 93–95), makes them attractive biomarker candidates for diagnosis or prognosis of complex diseases. However, the main challenges for profiling circulating miRNAs are biases introduced during pre-analytical and analytical steps that are described below.

### Biological Fluids and RNA Extraction

Analysis of circulating miRNAs is normally performed on peripheral blood plasma or serum, and to a lesser extent on sputum, urine, breast milk, saliva, semen, and cerebrospinal fluid (CSF). The choice of starting material can significantly impact the expression profiles that are generated; in particular, because each biofluid can be enriched for a distinct set of miRNAs (78, 96, 97).

The miRNA fraction in biological fluids typically represents a very low proportion of total RNA. Investigations using serum and plasma samples have demonstrated that different protocols for pre- and post-storage sample processing can impact the quality of subsequent RNA extraction (96, 98, 99). For example, collection of peripheral blood in heparin-coated tubes can inhibit downstream laboratory steps that are based on polymerase chain reaction (PCR) protocols (100, 101). In addition, special attention is required to avoid contamination with intracellular miRNAs originating from blood components such as platelets and erythrocytes, which can introduce significant bias in circulating miRNA expression profiles (96, 98, 99, 102). It is also important that circulating miRNAs with low GC content are not lost when performing phenol-based RNA isolation, a problem that can be overcome by using small RNA extraction kits customized for specific biofluids (103).

### Expression Profiling Methods for Circulating miRNAs

The biochemistry and molecular structure of miRNAs can cause difficulties for accurate transcriptional profiling and quantification (104, 105). Consequently, various established techniques for mRNA detection have been modified to improve miRNA detection, irrespective of tissue type (106–108).

Reverse transcription quantitative real-time PCR (RT-qPCR) is currently the most widely used method for miRNA profiling: it provides excellent sensitivity, high sample throughput, and the capacity for moderate multiplexing of targets (109). A number of strategies can be used with miRNA RT-qPCR, including (1) reverse transcription using stem loop primers as implemented in TaqMan™ MicroRNA Assays (110); (2) incorporation of locked nucleic acids (LNAs) (111) in primer sequences to reduce melting temperature (*T*_m_) differences in primer–target duplexes, as used for miRCURY LNA™ Universal RT microRNA PCR; and (3) approaches that enzymatically incorporate a poly(A) tail to miRNAs prior to the reverse transcription step, which facilitates hybridization with a poly(T) sequence linked to a universal reverse primer (109). Regardless of these technical developments, RT-qPCR-based methods cannot identify novel miRNAs and, importantly, special attention is required for the design of standardized internal controls (104, 112–114).

Hybridization-based methods normally rely on DNA capture probes that are immobilized on a microarray platform such that fluorescent signal intensities can be quantified to estimate expression of individual miRNAs. Commercially available and cost-effective microarray assays include miRCURY LNA™ microRNA Arrays (Exiqon), GeneChip^®^ miRNA Arrays (Affymetrix), and SurePrint miRNA Microarrays (Agilent) (115). However, due to lower specificity and reduced dynamic range compared to other methods, microarrays often require additional validation *via* RT-qPCR (116, 117). A relatively new hybridization-based method that does not require a PCR amplification step or direct labeling of target miRNAs is the nCounter^®^ miRNA Expression Assay developed by NanoString Technologies (118). This approach has comparable sensitivity to RT-qPCR, is high-throughput, and also facilitates multiplexing of up to 800 distinct miRNA variant targets in the same assay. miRNA profiling in serum (119), peripheral blood (120), and aortic tissue (121) provide examples of studies that have used this technology. However, like RT-qPCR, it is again important to note that hybridization-based profiling methods cannot be used to identify novel miRNA variants.

Unlike the methods discussed above, HTS technologies in the form of miRNA-seq can be used for discovery-focused global expression profiling of the whole miRNA transcriptome (miRNome) from a particular biological sample (122). In addition, miRNA-seq approaches can identify with high accuracy, novel mature miRNAs, sequence variants (specific isomiRs or particular miRNA family members), and also pre-miRNAs (37, 123, 124). The rapid adoption of HTS for miRNA profiling has been driven by significant increases in sample throughput, a wide range of laboratory methods for different applications, and a thriving ecosystem of open-source software for data analysis and interpretation (9, 125, 126). However, it is important to note that technical biases inherent to different sequencing technologies (e.g., Illumina^®^, ABI SOLiD^®^, and Ion Torrent™) may generate reads that are not bona fide miRNAs (5, 127, 128).

Finally, in addition to established methods described here, emerging biosensor approaches for miRNA profiling have been reviewed in detail elsewhere and are beyond the scope this review (129).

### Data Normalization

Transcriptomics experiments are characteristically “noisy,” therefore, appropriate normalization is critical to minimize technical variation that may compromise interpretation of results. A range of methods have been successfully used for this purpose in mRNA and intracellular miRNA transcriptomics. Manufacturers’ instructions for data normalization vary greatly depending on the platform used, and a consensus on how circulating miRNA data should be normalized is yet to emerge (114, 130).

There is significant debate concerning the optimal strategy for normalization of circulating miRNAs with RT-qPCR assays. Methods currently used include (1) normalization to small nucleolar RNAs as reference genes, (2) normalization to an external spike-in synthetic oligonucleotide, (3) normalization to specific miRNAs, and (4) the global mean normalization method (131). Most studies report the use of small nucleolar RNA genes as reference genes, such as the small nucleolar RNA, C/D box 44 gene (*SNORD44*), the small nucleolar RNA, C/D box 48 gene (*SNORD48*), and the RNA, U6 small nuclear 6, pseudogene (*RNU6-6P*). However, there is growing evidence that these genes may be unsuitable due to significant variability in expression among individual serum samples (130, 132, 133). Use of a synthetic RNA spike-in as a reference gene has also been criticized because this method only accounts for specific components of technical variation introduced, for example, during RNA extraction or reverse transcription (134). When identifying circulating miRNAs to serve as reference genes, normally those that do not vary significantly among biological replicates are selected. Marabita et al. (114) advise caution when selecting endogenous reference controls and recommend the use of data-driven approaches for this purpose. The global mean normalization method does not require a reference gene and appears to be the most robust option but should only be applied when simultaneously profiling hundreds of miRNAs (131).

Conflicting reports on the efficiency of methods for statistical normalization are also problematic for miRNA-seq data. Tam and collaborators evaluated a range of available methods and recommended the use of trimmed mean of *M*-values (TMM) (135) and upper quartile scaling (136) for count normalization in comprehensive miRNA profiling studies (137). Conversely, Garmire and Subramaniam (138) did not support the use of TMM but strongly recommended the application of locally weighted regression (139, 140) or quantile normalization (QN) (141) instead. However, a parallel benchmarking study published soon afterward came to the opposite conclusion, recommending TMM over QN (142). Finally, a rebuttal to Garmire and Subramaniam highlighted several drawbacks with their data analysis and evaluation of the TMM method (143).

In summary, it is imperative that rigorous independent benchmarking studies are performed to systematically evaluate normalization methods proposed for miRNA profiling. With these challenges in mind, in the next section we review studies that have assessed the usefulness of circulating miRNAs as biomarkers for selected bacterial and viral infections.

## Differential Expression of Circulating miRNAs in Specific Infectious Diseases

### Human Tuberculosis (TB)

Human TB, caused by *M. tuberculosis*, continues to be a significant global health problem with 9.6 million new cases and 1.5 million deaths in 2014 (144). Classical methods for TB diagnostics in clinical settings include smear microscopy and mycobacterial culture. The former is the most used test in middle- and low-income countries, but its sensitivity is highly variable (20–60%), and the latter can take up to 8 weeks to yield results (145).

Diagnostic tests based on molecular methods represent a significant improvement in turnaround time and accuracy. Nonetheless, most molecular-based platform assays in use today are costly and have not been designed to be used in lower tiers of the health-care system (145, 146). According to Pai and Schito, one of the highest priorities for TB diagnostics is the development of a point-of-care non-sputum-based test capable of detecting all forms of TB, including extra pulmonary TB. Improved methods for distinction between active and latent TB are also urgently required (146).

Human TB was one of the first infectious diseases to be targeted for development of new diagnostics based on circulating serum or plasma miRNAs. Using a human miRNA microarray platform (Exiqon miRCURY™ LNA), Fu and colleagues were able to detect 92 differentially expressed miRNAs in serum from patients with active pulmonary TB compared to healthy individuals (147). However, it is important to note that an appropriate correction procedure for multiple statistical tests was not used in this study. Notwithstanding this, RT-qPCR validation demonstrated that circulating miR-93* and miR-29a were significantly upregulated in serum from the TB cases. In addition, miR-29a was also shown to be differentially expressed in sputum samples from TB patients compared to healthy controls (HC). A follow-up study using the same miRNA expression microarray, but with sputum samples from active pulmonary TB cases and HC, also found that miR-29a was upregulated in sputum from TB patients (148). However, inconsistencies were observed between the results obtained for circulating serum miRNAs by Fu and coworkers and those obtained by Yi and colleagues for sputum miRNAs. In particular, the 2 sets of 10 miRNAs that showed the most increased or decreased expression in TB patients were different for each body fluid (147, 148).

Parallel work using a different miRNA expression platform (Applied Biosystems TaqMan^®^ Low Density Array Human MicroRNA Panel) and a comparable statistical approach identified a total of 97 differentially expressed miRNAs in serum samples from active pulmonary TB patients compared to HC (149). Following RT-qPCR validation and receiver operating characteristic (ROC) curve analysis, a panel of three miRNAs (miR-361-5p, miR-889, and miR-576-3p) was shown to differentiate TB patients from HC with moderate sensitivity and specificity. Further evaluation of the specificity of this panel of miRNAs for diagnosis of pulmonary TB was performed using RT-qPCR analysis of serum from pediatric patients infected with enterovirus, varicella-zoster virus, or *Bordetella pertussis*. All three miRNAs exhibited significant differences between the TB patient group and the other microbial infection groups, leading Qi and colleagues to propose this set of miRNAs as the starting point for a biosignature of human TB (149).

A comparative study of the diagnostic potential of a small panel of circulating serum miRNAs for pulmonary TB, lung cancer, and pneumonia was undertaken by Abd-El-Fattah et al. (150). Using RT-qPCR, these workers examined expression of four miRNAs (miR-21, miR-155, miR-182, and miR-197) in serum from pulmonary TB, lung cancer, and pneumonia patient groups compared to a HC group. They observed that all four miRNAs were significantly differentially expressed between lung cancer patients and HC, three miRNAs (miR-21, miR-155, and miR-197) distinguished pneumonia patients from controls, but only one miRNA (miR-197) was significantly differentially expressed between the pulmonary TB group and the control group.

Two independent miRNA-seq studies of circulating serum miRNAs for diagnosis of active pulmonary TB revealed distinct panels of miRNAs as potential expression biomarkers of disease. The first study (151) showed that six circulating serum miRNAs (miR-378, miR-483-5p, miR-22, miR-29c, miR-101, and miR-320b) could serve as a distinct biosignature of pulmonary TB compared to HC, and importantly, groups of pneumonia, lung cancer, and chronic obstructive pulmonary disease patients. Furthermore, ROC curve analysis demonstrated that a six-miRNA biosignature could discriminate pulmonary TB patients from HC with a sensitivity of 95.0% and a specificity of 91.8% (151). In the second study (152), miRNA-seq was used to identify a total of 30 circulating serum miRNAs that were differentially expressed (24 increased and 6 decreased) in active pulmonary TB patients compared to 3 different control groups (latent TB infection, BCG-vaccinated, and HC). However, only 1 of these 30 circulating serum miRNAs (miR-22) was also detected in the earlier study by Zhang et al. (151).

Natural killer (NK) cells are effector lymphocytes that represent an important component of the innate immune system; they are able to rapidly target and kill virus-infected and tumorigenic cells in the absence of antibodies (153). Zhang et al. (154) observed decreased expression of circulating serum miR-155 in TB patients when compared to HC. From a functional perspective, levels of miRNA-155 were also inversely associated with cytotoxicity of NK cells isolated from the TB patients, which suggested that miR-155 may be used as an indicator of NK cell activity in TB patients (154).

The human TB studies described in this review have used serum and sputum as a source of circulating miRNAs and multiple transcriptomics technologies (miRNA-seq, microarray, and RT-qPCR) and data normalization methods, which together may contribute to the discordance among the results obtained by different researchers. Table 1 provides summary information on circulating miRNA biomarker studies for diagnosis and prognosis of human TB.

**Table 1:** **Circulating microRNAs (miRNAs) profiled in selected human tuberculosis studies**.

Platform/assay	Biological fluid	Notable miRNAs detected (arrows indicate direction of expression)	Data normalization	Reference
miRCURY LNA array (Exiqon)	Serum and sputum	miR-93*↑, miR-29a↑	Median normalization	Fu et al. (147)
Gene Amp PCR system 9700 (Applied Biosystems)			U6 snRNA	
miRCURY LNA array (Exiqon)	Sputum	miR-3179↑, miR-147↑, miR-19b-2*↓, miR-29a↑	Median normalization	Yi et al. (148)
GeneAmp PCR System 9700 (Applied Biosystems)			U6 snRNA	
TaqMan Low Density array (Applied Biosystems)	Serum	miR-361-5p↑, miR-889↑, miR-576-3p↑	cel-miR-238	Qi et al. (149)
TaqMan RT-qPCR (Applied Biosystems)			miR-16	
7500 Real-Time PCR system (Applied Biosystems)	Serum	miR-197↑	SNORD68	Abd-El-Fattah et al. (150)
Solexa Small RNA-seq (Illumina)	Serum	miR-378↑, miR-483-5p↑, miR-22↑, miR-29c↑, miR-101↓, miR-320b↓	No information provided concerning miRNA-sequencing normalization method	Zhang et al. (151)
SYBR green RT-qPCR assay			miR-16	
Solexa Small RNA-seq (Illumina)	Serum	miR-516b↑, miR-486-5p↓, miR-196b↑, miR-376c↑	Total copy number of each sample was normalized to 100,000	Zhang et al. (152)
TaqMan RT-qPCR (Applied Biosystems)			cel-miR-238	
SYBR green RT-qPCR assay	Serum	miR-155↓	U6 snRNA	Zhang et al. (154)

### Sepsis

Sepsis is a subtype of systemic inflammatory response syndrome (SIRS), which is caused by an immune response triggered by various microbial infections. The causative agent is most commonly a bacterial pathogen, but it can also be triggered by infections involving fungi, viruses, or parasites (155). Sepsis is a major burden on health-care systems and of greatest concern in intensive care units (ICUs), where delayed diagnosis is a major cause of mortality. Consequently, in recent years there has been a concerted effort to develop circulating miRNA biomarkers for sepsis diagnosis and prognosis (156, 157).

Plasma levels of miR-150 have been shown to correlate with those of TNF-α, IL-10, and IL-18, which are important immune response markers. More specifically, the ratio of miR-150/IL-18 has been suggested as a useful indicator of sepsis (158). miR-150 was also shown to exhibit increased expression in plasma from septic shock patients and was an independent predictor of mortality (159). Contrary to these results, circulating serum miR-150 levels could not be used to differentiate between critical illness patients and healthy individuals. However, although circulating miR-150 had no association with common markers of inflammation, it was independently correlated with unfavorable prognosis for patients (160).

It has previously been proposed that decreased expression of circulating miR-146a serves as an indicator of sepsis in both serum (161) and plasma (162). In addition, miR-223, which also exhibits decreased expression in plasma during sepsis, has been shown to display a greater capacity to distinguish sepsis from non-infectious SIRS than miR-146a (161). However, using more stringent statistical methods, a recent study demonstrated that miR-146a and miR-223 neither exhibited differential expression in plasma samples of sepsis and septic shock patients nor were they correlated with markers of inflammation, disease progression, or mortality (159). In addition, a comprehensive animal and clinical study has demonstrated that miR-223 serum levels do not correspond to the presence of sepsis in murine models or in a large cohort of ICU patients and do not reflect clinical outcome for critically ill patients (163). Taken together, these results constitute good evidence that circulating serum miR-223 cannot be used as a biomarker for sepsis.

In a murine model of polymicrobial sepsis, circulating serum miR-133a, miR-155, miR-150, and miR-193b* displayed increased expression compared to baseline measurements (164). When extended to humans using large cohorts of ICU patients and HC, serum miR-133a also exhibited increased expression and displayed an increasing trend with disease severity. In addition, miR-133a was correlated not only with markers of inflammation and bacterial infection but also with renal and hepatic damage, cholestasis, and liver biosynthetic capacity. This work therefore supports further evaluation of miR-133a as a useful marker for the clinical state of critically ill patients (164). Another recent study using a murine model that was extended to human patients also revealed that miR-122 displayed increased expression independent of the presence of infection or sepsis in human ICU patients (165).

It has been shown that circulating serum miR-297 is upregulated in non-surviving sepsis patients when compared to survivors, whereas miR-574-5p is downregulated. In addition, the combination of sepsis stage, sequential organ failure assessment (SOFA) score and miR-574-5p expression was identified as an excellent predictor for patient survival from sepsis (166). Finally, another comprehensive study investigated aberrantly expressed serum miRNAs, demonstrating that a combination of four miRNAs (miR-15a, miR-16, miR-193b*, and miR483-5p) and three clinical indicators (SOFA score, acute physiology and chronic health evaluation score, and sepsis stage) can be used as a good predictor for mortality by sepsis (167).

Table 2 provides a summary of methodologies and notable miRNAs profiled for the sepsis studies discussed in this section.

**Table 2:** **Circulating microRNAs (miRNAs) profiled in selected sepsis studies**.

Platform/assay	Biological fluid	Notable miRNAs detected (arrows indicate direction of expression)	Data normalization	Reference
miRNA microarray (Agilent Technologies)	Plasma	miR-486↑, miR-182↑, miR-150↓, miR-342-5p↓	Median normalization	Vasilescu et al. (158)
RT-qPCR TaqMan MicroRNA assays (Applied Biosystems)			U6B snRNA	
RT-qPCR TaqMan MicroRNA assays (Applied Biosystems)	Plasma	miR-150↑, miR-146a NS, miR-223 NS	cel-miR-39	Puskarich et al. (159)
RT-qPCR miScript system (Qiagen)	Serum	miR-146a↓, miR-223↓	mmu-miR-295	Wang et al. (161)
RT-qPCR miScript system (Qiagen)	Serum	miR-133a↑	SV40	Tacke et al. (164)
RT-qPCR miScript system (Qiagen)	Serum	miR-122↑	SV40	Roderburg et al. (165)
GeneChip miRNA 1.0 arrays (Affymetrix)	Serum	miR-297↑, miR-574-5p↓	5S rRNA	Wang et al. (166)
RT-qPCR miRcute (Tiangen Biotech Company)				
Solexa Small RNA-seq (Illumina)	Serum	miR-193b*↑, miR-15↑, miR-122↑, miR-483-5p↑, miR-16↓, miR-223↓	U6 snRNA	Wang et al. (167)
RT-qPCR TaqMan MicroRNA assays (Applied Biosystems)				

### Viral Hepatitis

#### Hepatitis B

According to the WHO, an estimated 240 million people are chronically infected with hepatitis B virus (HBV), and more than 686,000 people die every year due to complications of HBV infection (168). This viral infection attacks the liver and presents as acute or chronic disease. In comparison to other regions of the world, sub-Saharan Africa and East Asia show high endemicity of HBV infection (169). Additionally, chronic hepatitis B (CHB) infection is a major cause of liver cirrhosis and hepatocellular carcinoma (HCC), and reliable indicators of disease progression are urgently needed.

It has been shown that the occurrence of specific circulating miRNAs in blood serum of HBV-infected individuals increases with disease severity: 37 miRNAs in HC, 77 in chronic asymptomatic carriers, 101 in CHB, and 135 in HBV-associated acute-on-chronic liver failure (170). Circulating serum miR-210 (171) and miR-124 (172) are among the miRNAs implicated as being increased in conjunction with disease severity. Markers for liver fibrosis in HBV-infected patients have also been examined, with miR-345-3p, miR-371a-5p, and miR-2861 reported as positive indicators of fibrosis, whereas miR-486-3p and miR-497-5p exhibited lower expression at all stages of fibrosis when compared to non-fibrosis CHB patients (173).

Many published studies have suggested groups of circulating miRNAs that could distinguish CHB patients at early stages of HCC from those without the presence of cancer, such as plasma miR-122, miR-223, miR-26a, miR-27a, miR-192, miR-21, and miR-801 (174); plasma miR-28-5p, miR-30a-5p, miR-30e-3p, miR-378a-3p, miR-574-3p, and let-7c (175); serum miR-222, miR-223, and mir-21 (176); serum miR-206, miR-141-3p, miR-433-3p, miR-1228-5p, miR-199a-5p, miR-122-5p, miR-192-5p, and miR-26a-5p (177); and exosomal serum miR-221, miR-222, miR-224, and miR-18a (178). Furthermore, miR-150 (179) and miR-18a (180) have been independently profiled in serum of HBV-HCC patients and found to exhibit significantly higher expression in these groups when compared to CHB samples.

An miRNA consistently reported in hepatitis infection studies is miR-122. Significant higher levels of this miRNA in plasma (181, 182) and serum (170, 183–185) samples of HBV-infected patients have been observed, and hence miR-122 abundance has been suggested as a potential disease signature. miR-122 abundance was also positively correlated with current markers of viral activity in HBV-infected patients (170, 185, 186), but conflicting reports have been published regarding its correlation to degree of liver injury (170, 181, 186). It is also important to note that miR-122 was significantly upregulated in a murine model for alcohol- and chemical-induced liver diseases (181) and non-alcoholic steatohepatitis (184), which might implicate it as an unreliable biomarker.

miR-122 was also investigated as a component of miRNA panels that aim to offer a more robust biosignature of hepatitis B. For example, an assessment of CHB and healthy children during a period of 6 years revealed that miR-122-5p, miR-122-3p, miR-99a-5p, and miR-125b-5p could be used to monitor pathological status (187, 188). Furthermore, the combination of miR-122, miR-let7c, miR-23b, and miR-150 was able to distinguish either HBV or occult HBV-infected patients from HC (189).

Other miRNAs have also been reported as potential biomarkers for HBV-infected patients: miR-375, miR-10a, miR-223, and miR-423 distinguished HBV-infected patients from HC, and the combination of miR-920 and miR-423 was able to differentiate between HBV- and hepatitis C virus (HCV)-infected individuals (190).

Table 3 provides a summary of methodologies and notable miRNAs profiled for the HBV infection studies discussed in this section.

**Table 3:** **Circulating microRNAs (miRNAs) profiled in selected hepatitis B studies**.

Platform/assay	Biological fluid	Notable miRNAs detected (arrows indicate direction of expression)	Data normalization	Reference
RT-qPCR TaqMan MicroRNA assays (Applied Biosystems)	Serum	miR-122↑, miR-16↑, miR-223↑, miR-19b↑, miR-20a↑, miR-92a↑, miR-106a↑, let-7b↑, miR-194↑	U6 snRNA	Ji et al. (170)
RT-qPCR TaqMan MicroRNA assays (Applied Biosystems)	Serum	miR-210↑	cel-miR-39	Song et al. (171)
RT-qPCR miRcute (Tiangen Biotech Company)	Serum	miR-124↑	5S rRNA	Wang et al. (172)
miRNA microarray (Agilent Technologies)	Plasma	miR-4695-5p↑, miR-486-3p↓, miR-497-5p↓	Quantile normalization	Zhang et al. (173)
SYBR Green I-based RT-qPCR with individual miRNA-specific primers (Applied Biosystems)	Plasma	miR-122↑	U6 snRNA	Zhang et al. (181)
Thunderbird SYBR qPCR mix (Toyobo, Japan)	Plasma	miR-122↑	No information provided	Zhang et al. (182)
SYBR Green PCR Master Mixture (Takara)	Serum	miR-122↑	miR-181a	Xu et al. (183)
RT-qPCR TaqMan MicroRNA assays (Applied Biosystems)	Serum	miR-122↑	No information provided	Waidmann et al. (186)
RT-qPCR miRNA arrays and individual assays (Exiqon)	Plasma	miR-122-5p↑, miR-122-3p↑, miR-99a-5p↑, miR-125b-5p↑	Global mean normalization, U6, and geometric mean normalization	Winther et al. (187)
TaqMan probe-based RT-qPCR (Applied Biosystems)	Serum	miR-122↑, miR-let7c↑, miR-23b↑, miR-150↑	Plant MIR-168	Chen et al. (189)
Solexa Small RNA-seq (Illumina)	Serum	miR-375↑, miR-10a↑, miR-223↑, miR-423↑	Plant MIR-168	Li et al. (190)
TaqMan probe-based RT-qPCR (Applied Biosystems)				

#### Hepatitis C

Similar to HBV, the HCV can also give rise to both acute and chronic infection, possibly leading to progressive liver disease, cirrhosis, and hepatocellular cancer. Considering that miR-122 is a liver-specific miRNA (191), it is therefore not surprising that the majority of circulating miRNA studies have focused on miR-122 as a potential biomarker for hepatic pathologies. As previously observed in HBV studies, expression of circulating miR-122 has been found to be significantly higher in chronic HCV patients when compared to healthy cohorts (192–195). Panels of miRNAs that contained miR-122 also resulted in positive indicators for the presence of either CHC (66) or HCV (196). Positive correlation with current markers of HCV infection has been seen for miR-122 (66, 192) and miR-122-5p (196). However, miR-122 levels were also elevated in non-alcoholic fatty liver disease patients (193), and decreased levels were observed in one study with advanced stage fibrosis CHC patients (197).

Interestingly, high levels of serum miR-122 may predict favorable virological responses to therapy in pretreatment pegylated IFN alpha/ribavirin (pegIFN/RBV) patients of Asian ethnicity (198), but not for those of African and Caucasian ethnicities (199).

In a longitudinal study using plasma samples from non-HCV-infected injection drug users who eventually acquired the infection, miR-122 and miR-885-5p were increased in abundance during acute infection, whereas miR-494 and miR-411 were decreased in expression. Also, in an independent cohort of individuals, all but miR-411 were validated (200). Furthermore, miR-122 and miR-885-5p levels remained elevated during viremia and returned to preinfection levels after infection resolution (200).

Considering other miRNA species, miR-571 has been associated with HCV-related cirrhosis progression (201); miR-20a and miR-92a serum levels were elevated in HCV-infected fibrosis patients, and miR–92a expression was significantly reduced after infection resolution (202); circulating serum miR-320c, miR-134, and miR-483-5p were shown to be significantly increased in expression for HCV-infected patients when compared to HC (203).

Early detection of HCC is also a major concern for HCV patients. Several published studies have investigated the usefulness of circulating miRNAs as a less-invasive diagnostic method. Serum levels of miR–16 were lower in HCC patients when compared to either HCV (204) or chronic liver diseases (CLD) groups (205). miR-21 serum levels were elevated in CHC and CHC-associated HCC patients, in comparison with HC (206). In plasma samples, miR-21 levels were also significantly higher for HCC patients when compared to chronic hepatitis (B or C types) and healthy groups (207). Lastly, miR-199a exhibited moderate power to distinguish HCC patients from CLD groups (205).

Although there are many published studies describing the use of circulating miRNAs as biomarkers of hepatitis B (Table 3) or hepatitis C (Table 4), specificity and lack of independent validation remain significant problems hindering adoption of circulating miRNAs as useful biomarkers for hepatitis.

**Table 4:** **Circulating microRNAs (miRNAs) profiled in selected hepatitis C studies**.

Platform/assay	Biological fluid	Notable miRNAs detected (arrows indicate direction of expression)	Data normalization	Reference
RT-qPCR TaqMan MicroRNA assays (Applied Biosystems)	Serum	miR-122↑	No information provided	Bihrer et al. (192)
RT-qPCR TaqMan MicroRNA assays (Applied Biosystems)	Serum	miR-122↑, miR-34a↑, miR-16↑	cel-miR-238	Cermelli et al. (193)
RT-qPCR TaqMan MicroRNA assays (Applied Biosystems)	Serum	miR-122↑, miR-192↑	Normalized for initial serum input	van der Meer et al. (194)
RT-qPCR TaqMan MicroRNA assays (Applied Biosystems)	Serum	miR-122↑	cel-miR-39	Wang et al. (195)
RT-qPCR TaqMan MicroRNA assays (Applied Biosystems)	Serum	miR-122↑, miR-155↑	cel-miR-39	Bala et al. (66)
miRNA PCR arrays	Serum	miR-122↑, miR-134↑, miR-424-3p↑, miR-629-5p↑	U6 snRNA	Zhang et al. (196)
Individual RT-qPCR assays				
RT-qPCR TaqMan MicroRNA assays	Serum	miR-122↑	cel-miR-39	Su et al. (198)
TaqMan RT-qPCR OpenArray chips	Plasma	miR-122↑, miR-885-5p↑, miR-494↓	Quantile normalization	El-Diwany et al. (200)
Individual TaqMan RT-qPCR assays			ath-miR-159a	
miScript miRNA PCR array	Serum and plasma	miR-20a↑, miR-92a↑	cel-miR-39	Shrivastava et al. (202)
Individual TaqMan RT-qPCR assays				
miRNA microarray (Agilent)	Serum	miR-134↑, miR-320c↑, miR-483-5p↑	Percentile shift normalization	Shwetha et al. (203)

### Other Infectious Diseases

#### Pertussis

Pertussis, also known as whooping cough, is a respiratory infection caused by *B. pertussis*. A panel of five circulating miRNAs was observed to be upregulated in serum samples from infected patients (miR-202, miR-342-5p, miR-206, miR-487b, and miR-576-5p), showing high sensitivity and specificity for differentiation of pertussis patients and HC. In addition, analysis of this miRNA panel in samples from patients with a range of other microbial infections (*M. tuberculosis*, enterovirus, varicella-zoster virus, mumps virus, and measles virus) demonstrated that the expression signature for pertussis disease was distinct and unambiguous (208).

#### Human Immunodeficiency Virus (HIV)-Associated Neurological Disorders (HAND)

Cognitive, motor, and behavioral impairments that affect individuals infected with the HIV are collectively referred to as HAND (209, 210). An miRNA pairwise approach has demonstrated the potential use of two pairs of plasma miRNAs as biomarkers for cognitive-impaired HIV-positive individuals: miR-495-3p in combination with let-7b-5p, miR-151a-5p, or miR-744-5p; and miR-376a-3p/miR-16-5p (211).

It is recognized that early detection of HAND would facilitate better treatment choices and fewer sequelae caused by neuronal damage. However, this phenomenon has been difficult to investigate in patient cohorts; therefore, it has required the use of animal models such as the macaque (*Macaca nemestrina*) simian immunodeficiency virus (SIV) model of HIV (212). A combination of six circulating plasma miRNAs (miR-125b, miR-34a, miR-21, miR-1233, miR-130b, and miR-146a) could be used to predict the development of central nervous system disease in a macaque/SIV model, when animal samples from pre- and post-infection were compared to HC (212). Expression of circulating miRNAs in CSF of HIV-encephalitis (HIVE) patients has been compared to HIV-positive patients without signs of HAND, and also to HIV-negative individuals. Overall, decreased expression of miRNAs was observed between HIV-positive and HIV-negative groups, whereas between HIVE and HIV-negative no changes in expression were observed. General increased expression was only observed when HIVE and HIV-positive groups were compared, with miR-19b-2*, miR-937, and miR-362-5p displaying the largest fold changes (213).

#### Hand, Foot and Mouth Disease (HFMD)

Human enterovirus 71 (EV71) and coxsackievirus A16 (CVA16) are the most common pathogens responsible for HFMD. More than 500,000 cases, including 176 fatal ones, have been reported in China since an outbreak in 2008 (214). Levels of eight circulating serum miRNAs (miR-148a, miR-143, miR-324-3p, miR-628-3p, miR-206, miR-140-5p, miR-455-5p, and miR-362-3p) were significantly higher in sera of patients with enteroviral infections (215). The combination of six miRNAs (miR-148a, miR-143, miR-324-3p, miR-628-3p, miR-140-5p, and miR-362-3p) generated a biosignature that could distinguish enteroviral patients and HC. In addition, a panel comprising miR-143, miR-324-3p, and miR-545 had moderate ability in discriminating patients infected with CVA16 from those with EV71 (215). Circulating exosomal miRNAs (miR-671-5p, miR-16-5p, and miR-150-3p) have also been observed to be differentially expressed in serum samples from both mild and extremely severe cases of HFMD when compared to that from healthy individuals (216). Lastly, a signature of eight miRNAs (miR-494, miR-29b-3p, miR-551a, miR-606, miR-876-5p, miR-30c-5p, miR-221-3p, and miR-150-5p) was identified in serum of children infected with EV71 (119). Furthermore, the results presented by Wang and collaborators suggested that upregulation of miR-876-5p is a specific response to severe EV71 infection.

#### Varicella

Varicella, commonly known as chickenpox, is caused by the varicella-zoster virus. Aberrant serum miRNA expression in non-vaccinated children that contracted varicella revealed a panel of five miRNAs (miR-197, miR-629, miR-363, miR-132, and miR-122) that could differentiate, with moderate sensitivity and specificity, varicella patients from HC, and also varicella patients from patients with three other microbial infections (*B. pertussis*, measles virus, and enterovirus) (217).

#### Influenza

Influenza A viruses are the causative agents of influenza in birds and mammals. A panel of 14 circulating miRNAs was observed to be aberrantly expressed in whole blood samples from patients infected with the H1N1 strain of influenza virus A (218). Further analyses showed that six of these miRNAs (miR-1260, miR-335*, miR-664, miR-26a, miR-576-3p, and miR-628-3p) had similar expression signatures in human A549 and Madin–Darby canine kidney (MDCK) cells infected with H1N1 *in vitro*. In addition, examination of MDCK supernatant exosomes indicated that only miR-576-3p was not detectable (218). Also, evaluation of serum samples from influenza A/H1N1 patients demonstrated that critically ill patients exhibited elevated expression of miR-150, when compared to those presenting a milder form of the disease (219).

Avian influenza A (H7N9) has been recently detected in China and was associated with fatal cases. Circulating serum miR-17, miR-20a, miR-106a, and miR-376c were significantly increased in expression for patients compared to healthy individuals (220).

### Infectious Diseases of Veterinary Importance

*Staphylococcus aureus* is most common causative agent of contagious bovine mastitis, which represents a significant economic burden to the dairy industry (221). Evaluation of milk exosomes from *S. aureus*-infected Holstein cows indicated miR-142-5p and miR-223 as potential biomarkers for this infection in mammary glands (222).

It is important to note that the use of age-matched subjects may be critical for experiments designed to detect circulating miRNAs as biomarkers of infection in young animals. Consequently, results from such experiments should be interpreted with caution since they may be confounded by expression of miRNAs associated with developmental processes. This issue has been addressed recently by Farrell and colleagues using serum samples collected from calves at preinfection and 6 months after infection with *Mycobacterium avium* subspecies *paratuberculosis* (MAP). Expression profiling of circulating miRNAs *via* miRNA-seq showed differential expression of miR-205 and miR-432, but a signature of infection could not be identified (223). On the other hand, analysis of biobanked bovine serum samples from experimental infections with MAP (stored at −20°C for 10–15 years) revealed that the circulating miRNA profile was remarkably similar in composition to the profile from fresh sera (<1 year at −80°C) (94). miRNAs have also been shown to correlate with season (summer, after the calves were born; fall, at weaning; and the following spring) when serum from *Mycoplasma bovis*-infected calves was profiled, but similar to the MAP studies described above a strong signature of infection was not observed (224).

Studies focusing on miRNAs in animals of veterinary relevance are still relatively few and most are restricted to cellular miRNAs. However, unlike human studies where cohort sizes may be limited, where age, gender, and ethnicity profiles may differ, and where patient histories cannot be readily assessed, veterinary studies can leverage sophisticated experimental designs that may help uncover nuances of circulating miRNA expression profiles that better represent disease status in infected individuals.

A summary of the results obtained for studies discussed in Sections “Other Infectious Diseases” and “Infectious Diseases of Veterinary Importance” are presented in Table 5.

**Table 5:** **Circulating microRNAs (miRNAs) profiled in selected infectious disease studies**.

Pathology	Platform/assay	Biological fluid	Notable miRNAs detected (arrows indicate direction of expression)	Data normalization	Reference
Pertussis (human)	RT-qPCR TaqMan Array Human miRNA panel (Applied Biosystems)	Serum	miR-202↑, miR-342-5p↑ miR-206↑, miR-487b↑, miR-576-5p↑	cel-miR-238	Ge et al. (208)
Varicella (human)	RT-qPCR TaqMan Array Human miRNA panel (Applied Biosystems)	Serum	miR-197↑, miR-629↑, miR-363↑, miR-132↑, miR-122↑	cel-miR-238	Qi et al. (217)
Influenza H1N1 (human)	miRCURY LNA microRNA Arrays (Exiqon)	Whole blood	miR-1260↑, miR-335↑*, miR-664↑, miR-26a↓, miR-576-3p↑, miR-628-3p↓	Normalized to endogenous controls and the spike-in control	Tambyah et al. (218)
	RT-qPCR TaqMan			18S rRNA	
Influenza A/H1N1 virus (human)	RT-qPCR TaqMan Array Human miRNA panel (Applied Biosystems)	Serum	miR-150↑	U6 snRNA	Moran et al. (219)
Avian Influenza A H7N9 (human)	RT-qPCR TaqMan Array Human miRNA panel (Applied Biosystems)	Serum	miR-17↑, miR-20a↑, miR-106a↑, miR-376c↑	cel-miR-238	Zhu et al. (220)
Hand, foot and mouth disease (human)	RT-qPCR TaqMan Array Human miRNA panel (Applied Biosystems)	Serum	miR-148a↑, miR-143↑, miR-324-3p↑, miR-628-3p↑, miR-140-5p↑, miR-362-3p↑	cel-miR-238	Cui et al. (215)
Hand, foot and mouth disease (human)	miRNA microarrays (Agilent Technologies)	Serum exosomes	miR-671-5p↓, miR-16-5p↑, miR-150-3p↓	Standard Agilent normalization	Jia et al. (216)
	RT-qPCR			miR-642a-3p	
Hand, foot and mouth disease (human)	nCounter^®^ miRNA Expression assays (NanoString)	Serum	miR-494↑, miR-29b-3p↑, miR-551a↓, miR-606↓, miR-876-5p↑, miR-30c-5p↑, miR-221-3p↓, miR-150-5p↓	Geometric mean of top 100 miRNAs	Wang et al. (119)
	RT-qPCR TaqMan assays (Applied Biosystems)			U6 snRNA	
Human immunodeficiency virus (HIV)-associated neurological disorders (human)	RT-qPCR microRNA panels (Exiqon)	Plasma	miR-151a-5p↑, miR-194-5p↑, miR-19b-1-5p↑	miR-23a-3p and miR-23b-3p	Kadri et al. (211)
HIV-encephalitis (human)	RT-qPCR microRNA panels (Exiqon)	Cerebrospinal fluid	miR-19b-2*↑, miR-937↑, miR-362-5p↑	miR-622 and miR-1266	Pacifici et al. (213)
*Staphylococcus aureus*-induced mastitis (bovine)	TruSeq small RNA sequencing (Illumina)	Bovine milk exosomes	miR-142-5p↑, miR-223↑	Upper quantile normalization	Sun et al. (222)

## Conclusion and Future Perspectives for Circulating miRNAs as Biomarkers of Infection

Robustness to adverse sampling and storage conditions, especially for body fluids, is the most compelling reason that circulating miRNAs have significant potential as ancillary minimally invasive biomarkers for a wide range of pathologies. Thus far, research work has focused on identifying biomarkers and/or biosignatures for several diseases; however, studies that use systematic validation and independent confirmation are still relatively rare. In this regard, inconsistent results have been described across multiple studies that may be attributable to underpowered experimental design, lack of validation in subjects with pathologies that elicit similar immune responses, absence of standardized reference genes for normalization, and inappropriate statistical methodologies (e.g., no correction for multiple testing). Therefore, throughout the workflow, from sample collection and handling to the downstream bioinformatics, standardization of analytical methods will be key to establishing circulating miRNAs as robust and reliable biomarkers in clinical settings.

## Author Contributions

CC and RS reviewed the relevant literature and drafted the initial manuscript. CC, RS, and DM prepared the figure. NN, KM, and JB contributed additional material to the manuscript. DM and SG supervised the study and oversaw preparation, editing, and revision of the manuscript. All the authors have read and approved the final manuscript.

## Conflict of Interest Statement

The authors declare that the research was conducted in the absence of any commercial or financial relationships that could be construed as a potential conflict of interest.
